# Looking to the past to see the future: mother–child future talk following memory sharing in three cultural communities

**DOI:** 10.3389/fpsyg.2024.1528977

**Published:** 2025-01-22

**Authors:** Jessie Bee Kim Koh, Qi Wang

**Affiliations:** Department of Human Development and Psychology, College of Human Ecology, Cornell University, Ithaca, NY, United States

**Keywords:** future talk, memory sharing, culture, mental time travel, self

## Abstract

The present study examined mother–child spontaneous future talk following memory sharing in three cultural communities. Seventy-one European American, 60 Chinese American, and 58 mainland Chinese mothers and their 3-year-old children discussed two past events at home, one positive and one negative. Chinese and Chinese American mothers and children were more likely than European American mothers and children to spontaneously engage in future talk following memory sharing. After discussing negative past events, Chinese and Chinese American mothers and children were more likely than European American mothers and children to engage in didactic talk that emphasized children’s adherence to moral standards, social norms, and behavioral expectations in the future. Conversely, European American mothers were more likely than the two groups of Chinese mothers to engage in autonomous talk that emphasized children’s preferences and opinions regarding the future. Findings are discussed in light of the influence of mother–child conversations as a cultural context on the development of mental time travel and a temporally extended self.

## 1 Introduction

Remembering the past is for the preparation for the future (Neisser, 1988; Pillemer, 2001; Tulving, 2002; Wang and Koh, 2015). By reflecting on our past experiences, we gain insights about who we are and learn lessons to guide our future actions. One interesting question is whether parents spontaneously draw connections between the past and future when they share memories with their children, a common activity observed across cultural communities (Miller et al., 2007; Wang, 2013). Parent–child conversations about the past model to children how to evaluate their experiences, what aspects of an event are important to remember, and why the past is worth remembering, thus playing a critical role in children’s developing autobiographical memory and sense of self (Fivush, 2013; Nelson, 2007; Wang, 2021). No study to date has examined whether and how parents spontaneously connect children’s past experiences to their future endeavors following memory sharing. This is important because, by extending the past into the future, parents may be helping children see the temporal and casual links between the past and future, understand the consequences of past actions, make future plans according to past experiences, and learn lessons to guide future behavior. The present study fills this important gap by examining mother–child future talk following memory sharing in European American, Chinese American, and Chinese families.

### 1.1 Mother–child future talk

Notwithstanding the paucity of research on mother–child conversations about the future, the few studies conducted to date provide an important context for understanding future talk in the family. It has been observed that it is a frequent occurrence for parents to talk about the future with their children as young as 2 or 3 years of age. For example, in studying the conversational exchanges between 10 mothers and their 2-year-olds when they were engaging in various routine activities, Lucariello and Nelson (1987) observed 38 episodes of past talk and 44 episodes of future talk. This finding suggests that future talk is at least as common as past talk, if not more. Furthermore, the context in which mothers and children engage in conversational exchanges affects the frequency of future talk: Lucariello and Nelson observed that 89% of future talk (and 84% of past talk) occurred in the context of routine activities, such as lunch, getting dressed in the morning, and bathing/getting ready for bed. Routine activities are familiar and “scripted” events, where children’s event knowledge or scripts can facilitate their engagement in future talk with their mothers.

As future talk occurs, how do mothers discuss the future with their children? Hudson (2002) has observed that mothers use a variety of temporal languages during future talk: They talk about general event knowledge in present tense, past events in past tense, and future events in future tense, and they also discuss future hypothetical events involving references to possible actions, predictions of what might happen, and preferences in relation to the future event. This contrasts with past talk during which most of the maternal utterances (e.g., questions, statements) are references to the past events in past tense and most of the temporal terms are used to indicate event sequences (e.g., before, after, next). In addition, mothers use conventional time markers (e.g., morning, hour, month, Monday, January, Spring) about twice as much in future talk than past talk. These findings suggest that the language mothers use to talk with their young children about the future is temporally complex, more so than when they talk about past events.

Furthermore, maternal styles in discussing the future influence children’s contributions to the future talk. Hudson (2006) has identified three maternal styles in future talk: (i) elaborative/advanced language, characterized by the use of elaboration and references to future events, possible actions, predictions, and temporal terms; (ii) past/general events, characterized by references to past events and general event knowledge; and (iii) repetitive prompts/preferences, characterized by the use of repetitions and prompts and references to preferences. During future talk with their 2.5-year-old children, mothers who used more elaborative/advanced language and who made more references to past/general events had children who were more elaborative when talking about the future. With their 4-year-olds, mothers who used all three styles more frequently had children who were more elaborative during future talk, with the greatest effect coming from the use of elaborative/advanced language (Hudson, 2006).

Taken together, the few extant studies that examined mother–child future talk suggest that talking about the future is common in the family, even with very young children. Mothers engage in future talk with their children in ways that are different from when they engage in past talk. Through the various ways of talking about the future with their children, mothers scaffold children’s participation in the conversation. Importantly, this suite of research has focused on examining mothers and children talking about the future versus the past. Whether mothers relate the past to the future in the same conversation remains unknown. Also, future events in these studies are generally neutral. In contrast, research has shown that past events that mothers and children discuss are often emotionally charged, and that mothers and children engage in memory conversations differently for positive and negative events (Bauer et al., 2005; Fivush and Wang, 2005; Sales et al., 2003). Thus, to examine mother–child future talk following memory sharing, it is important to consider the valence of the past events.

### 1.2 Event valence in mother–child conversations

Positive and negative life events serve different functions in connection to the self (Fivush and Wang, 2005; Fredrickson, 2013; Grysman and Hudson, 2013). Whereas reflecting on positive events is associated with identity exploration and self-growth (Merrill et al., 2015), reflecting on negative events provides unique opportunities for individuals to gain insights about oneself and learn lessons from the experiences (Adler et al., 2016; Köber and Habermas, 2016; Wang et al., 2010). Accordingly, research on mother–child memory sharing has revealed that mothers and children talk about positive and negative events in different ways: They discuss more frequently the causes of the experienced emotions and make more coherent and complete accounts of what happened when conversing about negative than positive events (Ackil et al., 2003; Burch et al., 2004; Fivush and Wang, 2005; Sales et al., 2003).

Furthermore, in studies by Bird and Reese (2006) and Reese et al. (2007) that included outcome measures related to the self, it was found that during the discussion of positive events, mothers’ uses of explanations and confirmations of positive emotions were associated with children’s self-esteem, and children’s evaluations of the positive events was associated with their self-consistency. Conversely, during the discussion of negative events, both mothers’ and children’s explanations of the causes of negative emotions were associated with children’s self-esteem and self-consistency. Mother–child pairs’ discussion about resolving the negative emotions through social contact was also associated with children’s self-consistency. Similarly, Wang et al. (2010) found that mothers’ and children’s use of internal state language during reminiscing of negative events, but not positive events, uniquely predicted children’s trait and evaluative self-representations. Explanations of internal states in the negative event context also predicted children’s trait and evaluative self-representations.

Collectively, the findings suggest that the valence of past events influences the ways in which mothers and children share memories, which can in turn have different implications for the self. In general, sharing negative memories appears to have a greater impact on children’s sense of self than sharing positive memories. Talking about a difficult experience, although a potentially challenging conversation, may allow parents and children to discuss negative thoughts and feelings outside the heat of the moment, resolve the negative affect and conflict, and further reflect on the consequences of past actions. Thus, memory sharing about negative events with children may be a particularly important context for parents to draw connections between the past and future. Furthermore, culture, as a macro-contextual factor, may also play a critical role in how parents engage their children in future talk following memory sharing.

### 1.3 Culture and mother–child conversations

Culture shapes parent–child memory sharing in ways that reflect different values and child-rearing goals (Wang, 2013, 2021). Extensive research has shown that during memory conversations, European American mothers and children often focus on the child’s thoughts, feelings, and desires in the past events, where the child is cast as the central character of the story. This is in line with the European American cultural emphases on individuality, autonomy, and self-expression. In contrast, Chinese and Chinese American mothers and children often engage in conversations focusing on social interactions and behavioral rules and expectations. This is in line with the Chinese cultural values of social connectedness, interpersonal harmony, and moral rectitude (Koh and Wang, 2021; Wang et al., 2010; Wang and Fivush, 2005).

Cultural differences in memory conversations are particularly salient in the discussion of emotionally negative events (Fivush and Wang, 2005; Koh and Wang, 2021; Miller et al., 2007, 2012; Wang et al., 2010). European American mothers frequently focus on discussing and resolving the child’s negative emotions in the past event and often downplay the child’s past wrongdoings so as to protect their self-esteem. In contrast, Chinese and Chinese American mothers frequently engage children in didactic conversations about the child’s transgressions, where they help children reflect on their past wrongdoings, convey to children moral rules and behavioral expectations, and often end the conversation with a didactic coda that has implications for the child’s future conduct. It is possible that the different cultural modes of conversing about the past will be reflected in the future take following memory sharing, in alignment with the respective cultural values and socialization goals.

### 1.4 The present study

The present study examined mother–child future talk following the memory sharing of emotional experiences in European American, Chinese American, and Chinese families with 3-year-olds. Given their frequent use of memory sharing to instill proper behavior conduct in children (Koh and Wang, 2021; Miller et al., 2012; Wang and Fivush, 2005), we predicted that Chinese and Chinese American mothers would be more likely than European American mothers to spontaneously extend the past conversations into the future. Furthermore, given their independent versus interdependent cultural orientations (Keller et al., 2004; Wang, 2013), we expected that European American mother–child pairs would talk more about future plans involving only the child, whereas the two Chinese groups would discuss more future plans involving the child as well as others. We also predicted that European American mother–child pairs would discuss children’s emotions as well as their preferences and opinions in relation to the future more so than the two Chinese groups. Conversely, we predicted that Chinese and Chinese American mother–child pairs would refer to social interactions as well as moral and social rules in their future talk more so than European American mother–child pairs. Lastly, we predicted that the cultural differences would be more evident in the future talk following the discussion of emotionally negative than positive events.

## 2 Methods

### 2.1 Participants

Participants included 71 European American and 60 first-generation Chinese American mothers-child pairs from a university town and suburban areas in upstate New York, and 58 Chinese mothers-child pairs from Beijing, China. Children were recruited through local nursery schools and by word of mouth to participate in a larger longitudinal study on early social cognitive development. The European American (37 boys, 34 girls), Chinese American (30 boys, 30 girls), and Chinese (33 boys, 25 girls) children had an average age of 35.49 (*SD* = 3.31), 35.00 (*SD* = 3.43), and 34.17 (*SD* = 2.54) months, respectively. All children were from middle–class families, and the majority of mothers (93% European American; 98.3% Chinese American; 77.6% Chinese) had at least a college education. Mothers provided informed consent for their children’s participation, and each child received a small gift.

### 2.2 Procedure

Two native female researchers in the respective cultures visited the participating families. English-Chinese bilingual researchers visited the Chinese American families, and mothers were asked to use the language that they usually spoke at home with their children. Materials were prepared in both English and Chinese, and a translation-and-back-translation procedure was carried out to ensure their equivalence in both literal and sense meanings. During the visit, mothers were first asked to play with their children so that children were engaged and relaxed. This was followed by a series of mother–child activities, including mother–child memory-sharing.

Specifically, mothers were asked to talk to their children about two specific, one-time events that they experienced with their children together, such as a trip to the science museum or amusement park. One event was emotionally positive to the child, and one was emotionally negative. Mothers were asked to select events that took place within the past 2 months so that the memories were fresh to children. Mothers were further asked to talk with their children in the manner that they usually spoke to each other at home. There was no time restriction so that the mother–child pairs could talk for as long as they wanted. The sequence of talking about the positive and negative events was counterbalanced across mother–child pairs within each sample. The mother–child memory sharing task took approximately 20 min and was video-recorded.

In addition, mothers filled out a shortened version of the Child Development Inventory (Ireton, 1992) designed to assess children’s language production and comprehension, as a potential covariate (Fivush, 2013; Nelson, 2007). The possible score ranges from 0 to 100 and Cronbach’s *α* = 0.93.

### 2.3 Coding future talk following mother–child memory sharing

Spontaneous future talk following each memory conversation was first identified and then coded following the categories described below. Coding was performed in the original languages. Propositions, defined as subject-verb constructions, were used as the coding unit (Fivush and Haden, 2005; Wang and Fivush, 2005). Each unique or implied verb in an independent clause constitutes a new propositional unit. For example, “We swung and swung” would be coded as one proposition, whereas “We swung and laughed” would be coded as two. Conversations of the positive and negative events were coded separately. The coding was done by using Noldus’s program, The Observer® 5.0, a digital coding system designed to score video data online, with the codes and scores directly entered into a computer (Noldus, 2003).

#### 2.3.1 Narrative volume

Two variables were adopted to measure the volume of spontaneous future talk following each memory conversation. The first was *conversational turns*, where the total number of turns taken by mothers and children, respectively, was counted. The second was *propositions*, where the total number of propositions in mothers’ and children’s respective utterances was counted. Following prior research (Fivush and Haden, 2005; Wang and Fivush, 2005), meaningful non-verbal responses, particularly nodding and shaking head (corresponding to “yes” and “no,” respectively) were counted for the codes.

#### 2.3.2 Narrative content

The specific content of spontaneous future talk following each memory conversation was coded into the following categories. Coding was mutually exclusive and exhaustive, namely, each proposition was coded into one and only one of the categories.

1. *Planning talk*: Two categories were coded regarding children’s future plans. The first was *individual (child) plan*, including mothers’ and children’s statements or questions concerning children’s future activities (e.g., M: What will you do tomorrow? C: I will buy an ice-cream). The second was *shared plan*, including mothers’ and children’s statements or questions about future activities involving both child and others such as mom and child’s friends or teacher (e.g., M: We will go back again; C: When are we going to Disney?).
2. *Emotion talk*: This category concerned child’s emotions in the future talk, counting mothers’ and children’s uses of emotion terms. Positive (e.g., happy, laughing) and negative emotion terms (e.g., sad, yelling) were counted separately.
3. *Autonomous talk*: This category reflected the child’s autonomy and agency regarding the future, including mothers’ statements or questions about children’s preferences and opinions regarding an object, person, or the event itself (e.g., M: Do you want to go to the zoo tomorrow?), and children’s expressions of personal preferences and opinions (e.g., C: I want to go to the zoo tomorrow).
4. *Relatedness talk*: This category tackled mothers’ and children’s utterances about social interactions and the role of others in the future activities (e.g., M: Mommy will buy you a toy tomorrow. C: I will give grandma a big hug).
5. *Didactic talk*: This category reflected the emphasis on moral rectitude in the child’s future behavior, including mothers’ and children’s statements or questions about moral standards, social norms, and behavioral expectations (e.g., M: Next time how should you behave when you are in school? C: Listen to the teacher and sit quietly).

#### 2.3.3 Event topics

For an exploratory descriptive analysis, the content topics (e.g., family outings, conflicts with others, scary things) of the past events discussed by mother–child pairs were coded (see Results section).

Two English-speaking research assistants coded the European American data, and two English-Chinese bilingual research assistants coded the Chinese American and Chinese data. Joint discussion sessions were held to ensure consistency in applying the same definitions of the codes to the three datasets. All coders were blind to the hypotheses of the study. For reliability, 20% of the data from each sample was coded. Kappas ranged from 0.86 to 0.92 for the European American sample, 0.80 to 0.95 for the Chinese American sample, and 0.87 to 1.00 for the Chinese sample. Joint discussion sessions were held to discuss and resolve any disagreement.

## 3 Results

Preliminary analyses revealed no systematic effects of gender or age on the coded variables; gender and age were thus not considered further. Children’s language score was found to have effects on some of the codes and was therefore included as a covariate in all subsequent analyses. Two Chinese American mother–child pairs did not engage in memory sharing because the children were uncooperative. They were thus excluded from further analysis. Results are presented in five sections: The first section focuses on the percentages of mother–child pairs who engaged in spontaneous future talk following memory sharing and the narrative volume. The next two sections focus on mothers’ and children’s future talk volume and content, respectively. In the fourth section, a descriptive analysis of how mother–child pairs talked about the future following different event topics is presented. This is followed by a final section with examples of conversational excerpts of the three cultural groups.

### 3.1 Future talk engagement

Not all mothers-child pairs spontaneously engaged in future talk following memory sharing. As such, the proportion of mother–child pairs who did so was first determined. Mothers who took at least one conversational turn to talk about the future following at least one of the memory discussions (i.e., positive and negative events) were considered to have engaged in future talk with their children. Future talk engagement was dummy coded, whereby mothers received a 1 if they engaged in future talk following memory-sharing and 0 if they did not. To examine cultural differences in future talk engagement, a binary logistic model was conducted and significant effects were followed up with focused comparisons. Mothers in the three groups differed in their likelihood to engage in future talk following memory sharing, *χ*^2^ (2, *N* = 187) = 13.92, *p* < 0.01, *φ* = 0.27, with Chinese American (66%, *N* = 39) and Chinese (74%, *N* = 43) mothers more likely than European American (41%, *N* = 28) mothers to do so. All focused comparisons were significant at *p*s < 0.01.

The subsequent analyses focused on the mother–child pairs who engaged in spontaneous future talk following memory sharing.

### 3.2 Narrative volume

Means and standard deviations for conversational turns and propositions by mothers and children who engaged in future talk are displayed in Table 1 by culture and event valence. These continuous variables were analyzed in a 3 (Culture: European American vs. Chinese American vs. Chinese) × 2 (Event Valence: Positive vs. Negative) repeated-measures ANOVA on mothers’ and children’s codes, separately, with culture as a between-subjects factor and event valence as a within-subjects factor.

**Table 1:** Means and standard deviations of narrative volume by culture and event valence.

Narrative volume	European American *M* (*SD*)	Chinese American *M* (*SD*)	Chinese *M* (*SD*)	Total *M* (*SD*)
Mothers				
Conversational turns				
Positive	2.04 (2.57)	1.86 (2.45)	1.57 (2.23)	1.79 (2.39)
Negative	1.54 (2.32)	2.25 (2.03)	2.26 (2.50)	2.07 (2.30)
Propositions				
Positive	3.39 (4.24)	2.72 (3.87)	2.60 (4.23)	2.85 (4.09)
Negative	2.61 (4.19)	4.00 (3.78)	4.07 (4.22)	3.66 (4.08)
Children				
Conversational turns				
Positive	1.64 (2.47)	1.44 (2.20)	1.31 (2.07)	1.44 (2.20)
Negative	1.29 (2.07)	1.58 (2.03)	1.88 (2.31)	1.62 (2.15)
Propositions				
Positive	1.75 (2.65)	1.56 (2.31)	1.36 (2.14)	1.53 (2.32)
Negative	1.29 (2.05)	1.61 (2.16)	1.93 (2.39)	1.65 (2.22)

For the mother–child pairs who engaged in future talk following memory sharing, there was no significant cultural difference in the conversational turns taken or propositions made by mothers and children, respectively. There was no significant difference either in the conversational turns and propositions in mothers or children between positive and negative event discussions.

### 3.3 Narrative content

The frequencies for the content codes of future talk were generally low, with means mostly <1 except for mothers’ autonomy and didactic talks. Mothers’ and children’s respective utterances were thus dummy coded and analyzed as categorical data. For each coded category, mothers and children received 1 if they gave any response in relation to that category and received 0 if no relevant response was made. The proportions of mothers and children who made emotion talk, including both positive and negative emotion terms, were too low to warrant reliable analyses (< 10%) and were therefore not considered further. Table 2 presents the percentages of mothers and children who responded in each content category by culture and valence. Using generalized estimating equations (GEE), each future talk content category was analyzed in a 3 (Culture: European American vs. Chinese American vs. Chinese) × 2 (Event Valence: Positive vs. Negative) binary logistic model for mothers and children separately, with culture as a between-subjects factor and event valence as a within-subjects factor. Significant omnibus effects were followed up with focused comparisons.

**Table 2:** Percentages of responses for content categories by culture and event valence.

Content category	European American (*N* = 28)	Chinese American (*N* = 39)	Chinese (*N* = 43)	Valence Total
Mothers				
Individual plan				
Positive	20	35	38	30
Negative	44	15	32	28
Culture total	30	24	35	
Shared plan				
Positive	47	41	32	40
Negative	17	4	16	10
Culture total	30	14	23	
Autonomous talk				
Positive	71	77	76	75
Negative	68	27	39	44
Culture total	70	53	59	
Relatedness talk				
Positive	64	59	52	58
Negative	66	47	64	59
Culture total	65	53	58	
Didactic talk (negative)	36	87	74	
Children				
Individual plan				
Positive	22	36	28	28
Negative	35	8	22	19
Culture total	28	18	25	
Shared plan				
Positive	16	18	13	15
Negative	6	0	3	0
Culture total	10	0	6	
Autonomous talk				
Positive	59	72	67	66
Negative	61	32	37	43
Culture total	60	52	52	
Relatedness talk				
Positive	18	22	26	22
Negative	14	10	20	15
Culture total	16	15	23	
Didactic talk (negative)	26	57	53	

#### 3.3.1 Planning talk

No significant effect was found for individual plan among mothers and children. There was a main effect of event valence on mothers’ discussion of shared plan, *χ*^2^ (1, *N* = 141) = 11.42, *p* < 0.01, *φ* = 0.28, whereby mothers were more likely to refer to shared plans in the future following positive event discussions than negative event discussions. The percentage of children who referred to shared plans during future talk was very low (< 10%) and were not analyzed further.

#### 3.3.2 Autonomous talk

There was a main effect of event valence on mothers’ autonomous talk, *χ*^2^ (1, *N* = 141) = 14.01, *p* < 0.001, φ = 0.32, qualified by a marginally significant Culture × Valence interaction, *χ*^2^ (2, *N* = 141) = 5.61, *p* = 0.06, φ = 0.20. European American mothers were marginally more likely than Chinese American and Chinese mothers to refer to children’s autonomy in the future following negative event discussions, *χ*^2^ (2, *N* = 72) = 5.81, *p* = 0.06, φ = 0.28, but not positive event discussions. Alternatively, both Chinese American mothers, *χ*^2^ (1, *N* = 53) = 14.42, *p* < 0.001, *φ* = 0.52, and Chinese mothers, *χ*^2^ (1, *N* = 55) = 6.04, *p* < 0.05, φ = 0.33, but not European American mothers, were more likely to refer to children’s autonomy in the future following positive than negative event discussions. There was a main effect of event valence on children’s autonomous talk, *χ*^2^ (1, *N* = 141) = 6.79, *p* < 0.01, *φ* = 0.22, whereby children of all groups were more likely to express their autonomy in the future following positive than negative event discussions.

#### 3.3.3 Relatedness talk

There was no cultural difference in mothers’ and children’s respective discussions with regards to children’s social interactions in future. There was also no difference in mothers’ and children’s respective discussions about children’s future social interactions following positive versus negative event discussions.

#### 3.3.4 Didactic talk

Cross-tabulation showed that the numbers of mothers and children who engaged in didactic talk following positive event discussions were low (none of European American mothers and children; 4 Chinese American mothers and 1 child; 1 Chinese mother and 1 child), as would be expected. Analysis for didactic talk thus focused on future talk following negative event discussions. Using generalized linear model, didactic talk was analyzed in a binary logistic model for mothers and children separately.

Mothers in the different cultures differed in their likelihood to engage in didactic talk following negative event discussions, *χ*^2^ (2, *N* = 72) = 9.55, *p* < 0.01, φ = 0.36, with Chinese American and Chinese mothers more likely than European American mothers to do so. All focused comparisons were significant at *p*s < 0.05. Children in the different cultures did not show a significant difference in their likelihood to engage in didactic talk, *χ*^2^ (2, *N* = 72) = 3.33, *ns*., φ = 0.22. Given the effect size, exploratory pairwise comparisons were conducted, which revealed a significant difference between Chinese American and European American children, *p* < 0.05, and a marginal difference between Chinese and European American children, *p* = 0.08. Chinese American and Chinese children were more likely than European American children to engage in didactic talk following negative event discussions.

### 3.4 Mother–child future talk following different event topics

The content topics (e.g., conflicts with others, scary things) of the past events discussed by the mother–child pairs were first collated. Past events that were discussed by at least 20% of the mother–child pairs by culture and event valence were then identified. The ways in which mothers and children talked about the future following these identified past events were then tabulated. Table 3 summarizes the findings by culture and event valence.

**Table 3:** Mother–child future talk following different past event topics.

Event topics	Percentage	Types of future talk: mothers	Percentage	Types of future talk: children	Percentage
Positive events					
European American (*N* = 28)					
Outings and activities	40	Autonomous talk	88	Autonomous talk	88
		Relatedness talk	63		
Holiday events	25	Relatedness talk	80	Autonomous talk	60
		Autonomous talk	60		
Relationships	20	Shared plan	75	Shared plan	50
		Relatedness talk	50		
Chinese American (*N* = 39)					
Outings and activities	58	Autonomous talk	79	Autonomous talk	71
		Relatedness talk	57		
		Shared plan	57		
Chinese (*N* = 43)					
Outings and activities	80	Autonomous talk	75	Autonomous talk	65
		Relatedness talk	45		
Negative Events					
European American (*N* = 28)					
Scary things	31	Autonomous talk	100	Autonomous talk	100
		Relatedness talk	50	Individual plan	50
		Individual plan	50		
Separation from caregiver	23	Relatedness talk	100	Autonomous talk	66
		Autonomous talk	66		
		Shared plan	66		
Chinese American (*N* = 39)					
Conflicts with parents (including scolding)	38	Didactic talk	100	Didactic talk	82
Child injuries/Medical issues	24	Didactic talk	71	Autonomous talk	57
		Autonomous talk	57	Didactic talk	43
Conflicts with siblings	21	Didactic talk	100	Didactic talk	50
		Relatedness talk	100		
Chinese (*N* = 43)					
Conflicts with parents (including scolding)	23	Didactic talk	86	Didactic talk	71
		Relatedness talk	43		
Child injuries/Medical issues	23	Relatedness talk	86	Didactic talk	57
		Didactic talk	71	Autonomous talk	43

The most frequently discussed past positive event was outings and activities for all three cultural groups. European American mother–child pairs also frequently talked about holiday events and relationships. Across all three cultural groups, mothers were most likely to engage in autonomous talk, followed by relatedness talk, when discussing the future following memory sharing about these positive events. Likewise, across the three groups, children were most likely to engage in autonomous talk when discussing the future following memory sharing about these positive events.

The most frequently discussed past negative events were scary things and separation from caregivers for the European American sample. Following memory sharing of these negative events, European American mothers were more likely to have autonomous talk and relatedness talk than other types of talks when discussing the future, and their children were also more likely to engage in autonomous talk than other types of talks. For the Chinese American sample, the most frequently discussed past negative events were conflicts with parents (including scolding from parents), child’s injuries/medical issues, and conflicts with siblings. Following discussion of these negative events, Chinese American mothers and children were more likely to engage in didactic talk than other types of talks when discussing the future. Similarly, for the Chinese sample, the most frequently discussed past negative events were conflicts with parents and child’s injuries/medical issues. Following discussion of these past negative events, Chinese mothers and children were more likely to engage in didactic talk than other types of talks when discussing the future. In addition, Chinese mothers were also more likely to engage in relatedness talk than other types of talks.

### 3.5 Conversational excerpts

Example conversational excerpts of the three cultural groups are provided below. They illustrate the findings that following discussing negative past events, Chinese and Chinese American mother–child pairs often engaged in didactic talk to emphasize to children moral rules and behavioral expectations in the future, whereas European American mother–child pairs often engaged in autonomous talk to emphasize children’s preferences and opinions regarding the future.

**Chinese mother–child pair:**

Mother: You cried. You shouted. Did I pay attention to you?

Child: No.

Mother: No. How long did you shout? Very long, right?

Mother: Why were you angry?

Child: I did not keep my toys.

Mother: Didn’t keep toys. What else? Why did mom not pay attention to you? Was it because you shouted at me?

Child: Yes.

Mother: Yes. Will you shout at me again next time? Is it right to shout at me?

Child: Not right.

Mother: Not right. You should also keep your own toys, right?

Child: Yes.

**Chinese American mother–child pair:**

Mother: Why didn’t you want to eat today? Mom scolded you, right?

Child: Yes.

Mother: Dad also scolded you. What happened after that?

…

Mother: Where did you have your time-off?

Child: In the small room.

Mother: In the small room. Did you cry?

Child: Yes.

…

Mother: Next time, will you eat properly?

Child: Yes.

Mother: Yes?

**European American mother–child pair:**

Mother: When we went skiing, do you remember?

…

Mother: What happened that made it scary?

Child: Fell down…

Mother: That’s right. You slipped over, didn’t you?

Child: Yes.

…

Mother: And you were scared.

Child: Yes.

…

Mother: … Do you want to ski again next year?

Child: No.

Mother: No?

## 4 Discussion

Using the past to inform the future is an essential aspect of memory function (Neisser, 1988; Pillemer, 2001; Tulving, 2002; Wang and Koh, 2015). The present study is the first we know of to examine spontaneous mother–child future talk following memory sharing. It is further situated in the cultural context, examining conversations between European American, Chinese American, and Chinese mothers and their 3-year-old children. Findings showed that specific to this form of future talk, there were both cultural differences and similarities. The findings provide important insights regarding the ways in which mothers and children extend their conversations about past experiences into the future and have implications for children’s developing mental time travel and sense of self.

Consistent with our hypotheses, spontaneous future talk following memory sharing was found to be more common in Chinese and Chinese American mother–child pairs than European Americans. A closer examination on the future talk content revealed that the two groups of Chinese mothers and children were more likely than European American mothers and children to engage in didactic talk that emphasized children’s adherence to moral standards, social norms, and behavioral expectations, particularly following discussions about past negative events. Exploratory qualitative analyses further showed that this cultural difference was especially salient when the past event involved a prior conflict with parents (e.g., parental discipline) - the most discussed negative event topic in the two Chinese groups. It appears that Chinese and Chinese American mothers frequently use future talk following memory sharing to remind and teach children the proper ways of behaving in the future and, in turn, minimizing future social disharmony. Accordingly, the two groups of Chinese children also talked about how they would self-regulate and behave properly in the future. These findings suggest the greater usage of future talk for learning lessons and moral socialization in Chinese than European American families, in line with the Chinese cultural emphasis on moral rectitude and behavioral control (Koh and Wang, 2021; Miller et al., 2012; Wang and Song, 2014). Interestingly, spontaneous didactic talk is also frequently observed in naturally occurring, everyday conversations between caregivers and children in Chinese-heritage families, often triggered by ongoing activities, which may reflect the “opportunity education” principle of Chinese socialization (Miller et al., 2007, 2012; Wang, 2013).

In contrast, although European American mother–child pairs did not spontaneously engage in future talk as much as Chinese and Chinese Americans did following memory sharing, European American mothers were more likely than the two groups of Chinese mothers to have autonomous talk focusing on their children’s preferences and opinions with regards to the future, particularly following discussions of past negative events. It seems that even in the context of sharing negative memories, European American mothers link the child’s past experiences to their agency and autonomy in their future endeavors. This is in line with the European American cultural emphasis on individuality and personal choice (Keller et al., 2004; Wang, 2013). Interestingly, the most commonly discussed negative events among European American mother–child pairs involved scary things and separation from caregivers. Mothers’ engagement in autonomous future talk in this context may be encouraging children to cope with fear and separation from loved ones by taking control of the situation (e.g., what children think or prefer to do in face of such situations), in line with the emphasis on active coping in European American culture (Fabes et al., 2002; Yang et al., 2020). Similarly, European American children also expressed their future autonomy and agency following conversations about past fears or separations.

Thus, mother–child future talk following memory sharing models to children the important link between the past and the future in ways that reflect cultural values and socialization goals. Chinese and Chinese American mothers focus on instilling in children proper behaviors in the future to maintain social and familial harmony. By doing so, they are co-narrating with their children a temporally extended self that is interdependent, socially connected, and adhering to social norms and moral rules. In contrast, European American mothers focus on what children would like to see happening and how they would feel about it. By doing so, they are co-narrating with their children a temporally extended self that is independent, autonomous, and agentic. By age 3, children appear to have already internalized their mothers’ values into their ways of thinking about the future. Notably, the cultural differences were more salient in future talk following negative than positive event discussions, which supports the notion that conversing about negative events is a particularly sensitive setting for children to learn critical cultural messages (Fivush and Wang, 2005; Koh and Wang, 2021; Miller et al., 2007, 2012; Wang et al., 2010). Furthermore, the ways in which European American and Chinese mothers and children talk about the future are consistent with how they talk about the past as revealed in prior research (Koh and Wang, 2021; Miller et al., 2012; Wang and Fivush, 2005). Such similarities suggest that parent–child memory sharing and future talk both serve as an important socialization context to convey to children cultural values and expectations and together facilitate the development of a temporally extended self that reflects cultural priorities.

There were also important cultural similarities in future talk following memory sharing. The three cultural groups of mother–child pairs took similar numbers of conversational turns and produced similar numbers of propositions, which indicate that they engaged in future talk in similar interactive ways and lengths. Also, regardless of culture, mothers were more likely to discuss shared future plans following positive than negative event discussions. This is in line with the notion that memory sharing about positive events serves to maintain and strengthen parent–child bonds and further creates a sense of shared history (Sales et al., 2003; Fivush and Wang, 2005) that is then extended into the future. Furthermore, Chinese and Chinese American mothers were more likely to engage in autonomous future talk following positive than negative event discussions, although European American mothers did not show this valence-dependent difference. Children of all groups were also more likely to engage in autonomous future talk following positive than negative event discussions. Together, the current findings provide evidence for the different functions of positive and negative memories in informing the future: While positive experiences often entail continuity, agency, and growth, negative experiences call for change and redemption (Adler et al., 2016; Fivush and Wang, 2005; Köber and Habermas, 2016; Merrill et al., 2015). In addition, mothers of all cultures similarly engaged in relatedness future talk following memory sharing, which suggests that, just like past talk (Fivush, 2013; Nelson, 2007; Kulkofsky et al., 2009), future talk also serves as an important context to facilitate parent–child bonding and social connections.

Although the present study yielded original findings, there are some important limitations that can inform future research. Given that we focused on mother–child spontaneous future talk following memory sharing, rather than full-fledged mother–child conversations about the future, the number of mother–child pairs engaging in future talk, the length of future talk, and the range of content topics discussed were limited as a result. This also led to low frequencies in certain narrative codes and reduced statistical power. Future studies may recruit larger samples and also consider extending observation periods, tracking mother–child interactions in natural settings, or employing experimental designs. Notably, there has been a surge of research that examines children’s episodic future thinking (e.g., Atance, 2008; Bélanger et al., 2014; Busby and Suddendorf, 2005; Wang et al., 2014, 2024), but the social mechanisms underlying its development have rarely been investigated. Future studies may relate mother–child talk about future events to children’s episodic future thinking skills. In addition, the present study focuses on conversations between mothers and very young children, where mothers tend to lead the conservation. The active contributions of children might thus be underestimated. Future research should examine how children of different ages independently link their past experiences to future activities and what factors (including culture) influence this process (Suddendorf, 2010; Wang and Koh, 2015). Finally, the current samples were relatively homogenous within groups (i.e., all middle class, mostly highly educated) and thus not representative of the broader populations from which they were drawn. Future research should include parents and children from more diverse samples, as well as cultural communities that have not been previously studied, which can enrich our understanding of the critical role of culture in shaping family narrative practices and the use of memory for future preparation.

In conclusion, mothers and children in three cultural communities spontaneously talk about the future following memory sharing. Whether and how they engage in future talk reflect the values and socialization goals prioritized in their culture. Such conversations are important in modeling to children how to extend the past into the future through temporal and casual connections, plan for the future according to past experiences, and learn lessons to guide future behavior, in line with cultural expectations. They may thus facilitate the development of mental time travel and a temporally extended self.

## Data Availability

The raw data supporting the conclusions of this article will be made available by the authors, without undue reservation.
